# *RPS12* and *UBC4* Are Related to Senescence Signal Production in the Ribosomal RNA Gene Cluster

**DOI:** 10.1128/mcb.00028-22

**Published:** 2022-04-06

**Authors:** Shuichi Yanagi, Tetsushi Iida, Takehiko Kobayashi

**Affiliations:** a Laboratory of Genome Regeneration, Institute for Quantitative Biosciences (IQB), The University of Tokyo, Bunkyo-ku, Tokyo, Japan; b Collaborative Research Institute for Innovative Microbiology, The University of Tokyo, Bunkyo-ku, Tokyo, Japan

**Keywords:** ribosomal RNA gene (rDNA), life span, budding yeast, genome instability, senescence, noncoding transcription

## Abstract

Genome instability causes cellular senescence in many organisms. The rRNA gene cluster (rDNA) is one of the most unstable regions in the genome and this instability might convey a signal that induces senescence in the budding yeast. The instability of rDNA mostly depends on replication fork blocking (RFB) activity which induces recombination and gene amplification. By overexpression of Fob1, responsible for the RFB activity, we found that unstable rDNA induces cell cycle arrest and restricts replicative life span. We isolated yeast mutants that grew normally while Fob1 was overexpressed, expecting that some of the mutated genes would be related to the production of a “senescence signal” that elongates cell cycle, stops cell division and finally restricts replicative life span. Our screen identified three suppressor genes, *RPS12*, *UBC4*, and *CCR4*. Replicative life spans of the *rps12* and *ubc4* mutants were longer than that of wild-type cells. An increase in the levels of extrachromosomal rDNA circles and noncoding transcripts, known to shorten replicative life span, was observed in *ubc4* and *rps12* respectively, while DNA double strand-breaks at the RFB that are triggers of rDNA instability were reduced in the *rps12* mutant. Overall, our observations indicate that Rps12 and Ubc4 contribute to the connection between rDNA instability and replicative life span.

## INTRODUCTION

Genome instability is a major senescence promoting factor in many organisms (1). Mutations in genes for DNA repair and genome stability are known to reduce life span (2). This suggests that damaged DNA conveys a “senescence signal” that induces cell cycle elongation and arrest to affect replicative life span. Factors involved in triggering senescence might be double-strand breaks (DSB), noncoding transcription, resection, the presence of single-strand DNA, recombinational events, etc. And maybe onset of senescence is not determined by a single factor and that it is the accumulation of DNA damage that makes continuation of replication impossible. Such damage and instability mainly occur at fragile sites in the genome.

The rRNA gene cluster (rDNA) is one of the largest fragile sites in the genome (for review, see reference 3). In the budding yeast Saccharomyces cerevisiae, the rDNA forms a large tandem repeat structure that can go through cycles of contraction and expansion. Due to spontaneous deletion and subsequent gene-amplification, the number of repeats, the rDNA copy number, is dynamic (4). This instability of rDNA in S. cerevisiae is known to affect replicative life span, suggesting that the site is a source of a senescence signal (5).

**FIG 1:**
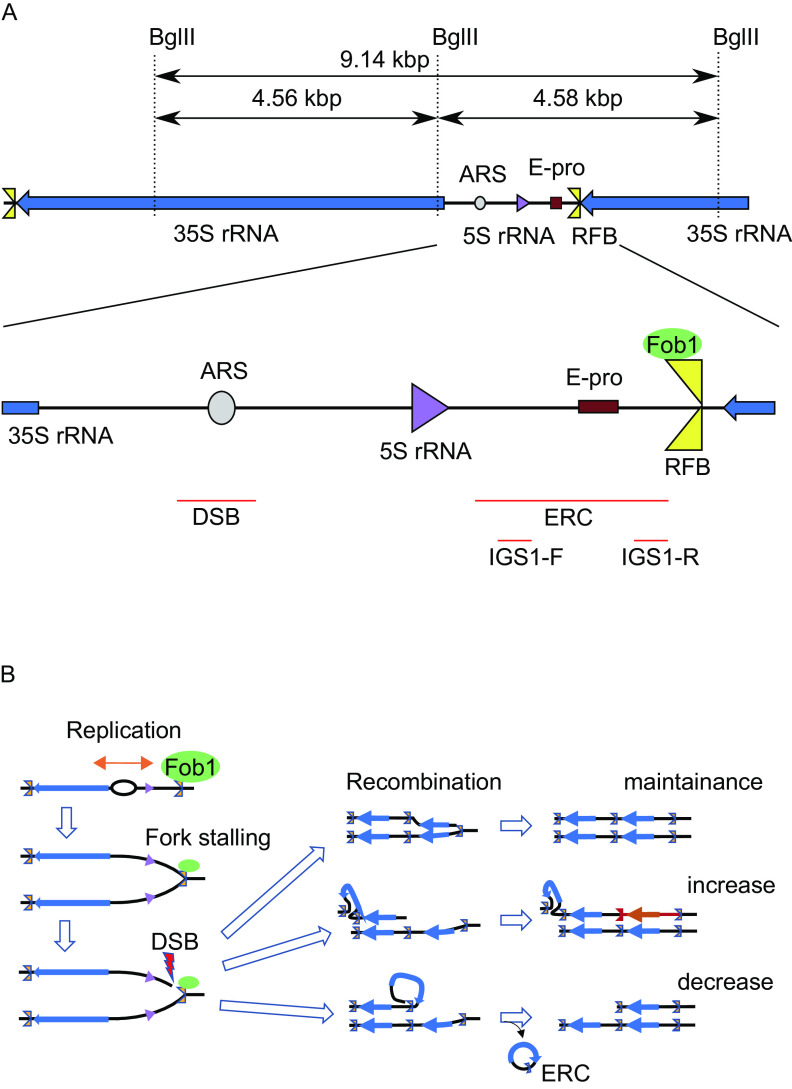
Schematic representation and recombination of rDNA. (A) Schematic representation of the yeast rDNA locus. BglII recognition sites (dashed lines), detectable restriction fragments and positions of probes (red bars) used for Southern blot analysis of Fig. 2 and 6 (ERC), or Fig. 8 (DSB) and for Northern analysis of Fig. 7 (IGS1-F and IGS1-R), are indicated. (B) Overview of the consequences from Fob1 dependent fork arrest. DSB are either repaired by recombination or lead to a change in copy number via unequal sister-chromatid recombination or the formation of ERCs.

The fragility of rDNA is mainly due to its structure of tandem repeated rDNA units. The budding yeast S. cerevisiae has ∼150 rDNA copies on chromosome XII that produce a huge amount of rRNA, which forms ∼60% of total RNA in a cell (4, 6). By accidental deletion, the rDNA locus can lose copies of an rDNA unit. To recover from this and maintain an adequate copy number to meet the huge demand for rRNA, the yeast uses a gene amplification system (4). In this system, the DNA replication fork barrier (RFB) site in the rDNA has a central role (7, 8) (Fig. 1). The RFB is located at the 3′-end of the 35S rRNA gene and arrests the replication fork by means of the RFB-associating protein Fob1 to prevent a run-in with 35S transcription (9, 10).

In the absence of Fob1-activity, neither amplification nor copy number alteration is detected (4). Moreover, DSBs at the RFB are detected in an arrested-fork dependent manner (11–14). Therefore, the Fob1/RFB interplay creates a recombination hot spot linked to gene amplification. Overall, as a result of recombination, the rDNA becomes an unstable site where amplification and contraction occur (for a review, see Ref. 15).

S. cerevisiae has been used as a model organism to study senescence, life span and aging (16). When a yeast cell proliferates, it divides into a large mother cell and a small daughter cell, that initially appears as a bud. A mother cell, from which about 20 daughter cells detach through its life, finally stops dividing and dies (16). Replicative life span is defined as the number that a mother cell can divide before it dies. In the last several cell divisions, detachment of the bud is delayed, the doubling time gets longer, and the daughter cells become bigger than usual (17–19). This phenotype is defined as senescence in the organism. In case of the budding yeast, a senescent cell with the cell cycle elongated divides several times and dies. In the *fob1* mutant, the replicative life span is extended (20, 21). In contrast, overexpression of *FOB1* shortened the replicative life span (22). These findings suggest that the replication arrest and/or the following rDNA recombination events produce senescence signals that restrict replicative life span, that is the number of cell-divisions of the mother cell (23).

Noncoding transcription is also related to rDNA recombination and replicative life span (5, 24) . A noncoding promoter, E-pro (Expansion promoter) was identified as an essential region for rDNA amplification (25). E-pro driven transcription removes cohesin from the surrounding region and enhances unequal sister-chromatid recombination that leads to re-replication which can increase the rDNA copy number (Fig. 1) (11, 24). In strains with a normal copy number, the transcription from E-pro is repressed by a histone deacetylase, Sir2 (24). When the rDNA copy number is reduced, however, an excess of UAFs (upstream activating factors for 35S rDNA transcription) are formed. UAFs that no longer can bind rDNA start to repress *SIR2* and by reducing Sir2 levels, enable the activation of E-pro, which enhances the rate of recombination in nearby rDNA (26). Therefore, in the absence of Sir2, E-pro is highly activated resulting in increased rDNA instability due to frequent copy number alteration and the replicative life span is shortened to about half of that of the wild type (27). These observations support the idea that Fob1/RFB dependent fork arrest and/or subsequent recombination events produce a senescence signal that finally restricts replicative life span.

Not only the putative senescence signal, but also other factors are known to promote senescence. One of the rDNA related factors are circular molecules that pop out from rDNA by recombination (28). These molecules, called ERCs (extra-chromosomal rDNA circles), accumulate in the mother cell during cell divisions and are supposed to titrate factors critical for viability (28). In fact, exponential accumulation of ERCs in an old mother cell is observed by single cell imaging (17).

Although the ERC accumulation has a strong impact on cell viability, the phenomenon seems to be specific to budding yeast. By manipulation of the replication initiation sequence in the rDNA we have created a strain in which the replication rate of ERC was decreased, and ERC accumulation was not prominent (29). This had, however, an opposite effect on replicative life span. In the strain with reduced ERC formation, rDNA instability was increased, and the replicative life span was shorter than that of the wild type. Such an increase of rDNA instability was also observed in the absence of Clb5 activity, which leads to reduced efficiency of replication initiation in rDNA (30). Thus, initiation of replication appears to be a determinant for maintaining rDNA stability rather than the secondary effect, formation of ERCs. These findings suggest that senescence in budding yeast can also be driven by genome instability like in mammalian cells.

To identify genes linked to the generation of a senescence signal that is produced by Fob1/RFB-dependent fork arrest, we screened for point mutants in which the growth inhibition caused by overproduction of Fob1 had been suppressed. As the result of our screen, three suppressor genes, *RPS12*, *UBC4*, and *CCR4*, were isolated that carried mutations causing single amino acid changes. Rps12 is a non-essential ribosomal component of the 40S subunit. The *rps12* deletion mutant is known to have a longer replicative life span although the molecular mechanism is not known (31). Ubc4 is a ubiquitin-conjugating enzyme (E2), related to protein degradation (reviewed in Ref. 32) and, together with its paralog Ubc5, implied in the control of various processes through interaction with various E3 ligases such as APC (22, 33) and Tom1, which controls the levels of unincorporated ribosomal proteins (34). Cells that do not produce Ubc4 have a longer chronological life span, another aspect of yeast aging (for replicative life span and chronological life span, see Ref. 35). These reports indicate that our screening could have been working as expected, at least to some extent. Ccr4 is a component of the CCR4-NOT complex that regulates gene expression and degradation of RNA (reviewed in Ref. 36). In contrast to the other two mutants, the *ccr4* deletion mutant has very unstable rDNA due to a highly activated E-pro (37), which, like *FOB1* overexpression, would lead to shortening of replicative life span. Possibly, the absence of Ccr4 only indirectly affects rDNA stability, so that the mutation might not directly be involved in the senescence pathway. We further analyzed the *RPS12* and *UBC4* suppressor mutants from the screen and found that *rps12-G77D* and *ubc4-P119L* bear features associated with the production of a senescence signal.

## RESULTS

### *FOB1* overexpression shortens replicative life span and inhibits growth.

**FIG 2:**
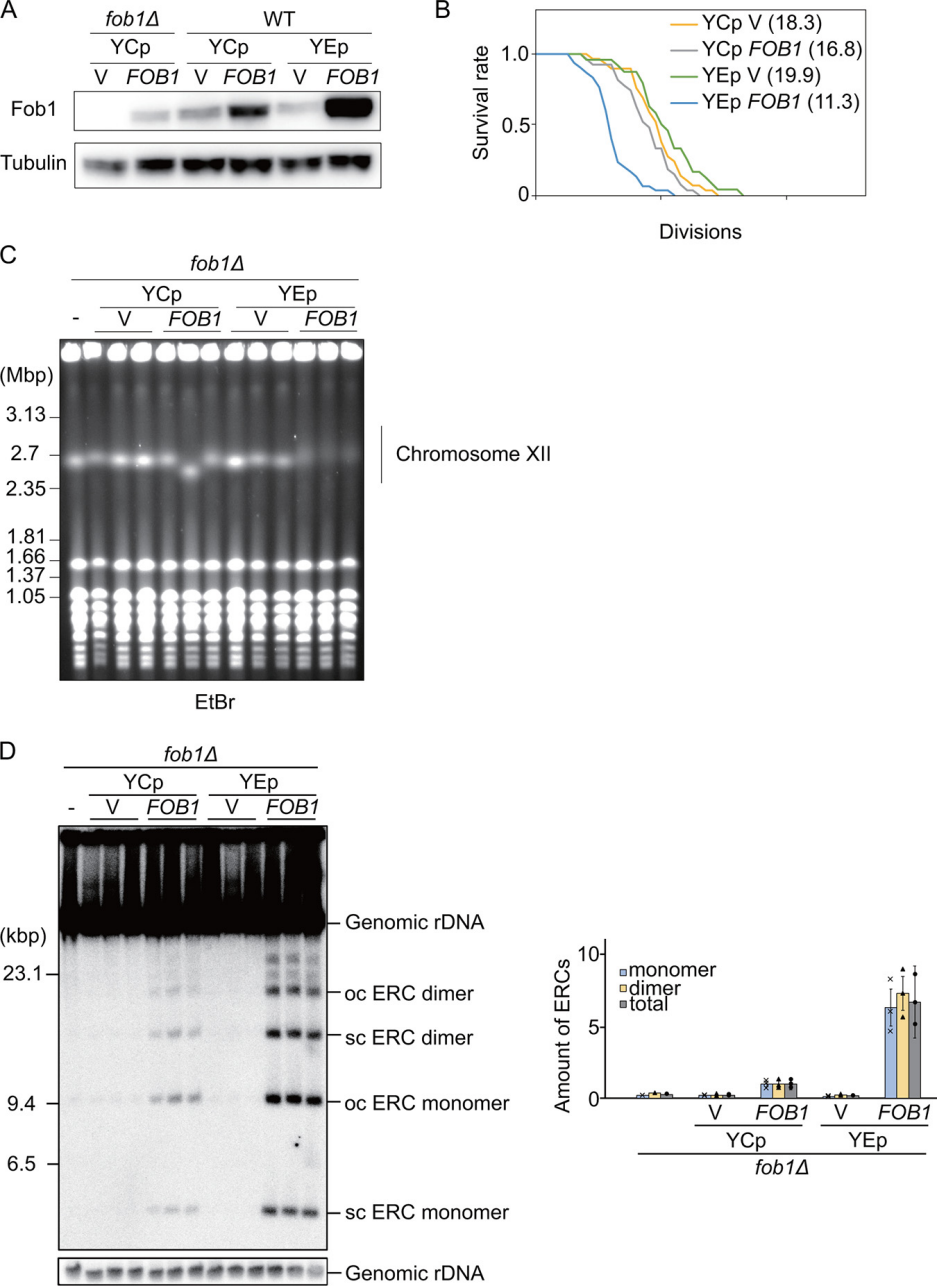
Characterization of *FOB1* overexpression from episomal plasmids (∼10-fold increase). (A) Fob1 levels in strains in relation to *FOB1* copy number. Fob1 and α-tubulin, a loading control, were detected by Western blotting analysis using anti-Fob1 and anti-tubulin antibodies. (B) Survival curves for replicative life span. The number of daughter cells that budded from each mother cell was counted for wild-type cells (WT) harboring a single-copy plasmid (YCp) or multi-copy plasmid (YEp) with or without *FOB1* gene. Numbers in brackets indicate the mean replicative life span with the number of mother cells counted, *n* = 29 (YCp), 27 (YCp *FOB1*), 24 (YEp), and 30 (YEp *FOB1*). (C) Stability of rDNA in relation to *FOB1* copy number. *fob1Δ* cells (lane -) were transformed with single- (YCp) or multi-copy (YEp) plasmids without (V) or with the Fob1 gene (*FOB1*). Genomic DNAs separated by pulsed-field gel electrophoresis as described in Materials and Methods, were visualized by staining with ethidium bromide. (D) Extrachromosomal rDNA circles in relation to *FOB1* copy number. ERCs in cells described for (A) were detected by Southern hybridization (left panel), quantified with respect to the amount of genomic rDNA and normalized to wild type (WT) (right panel). The form of ERCs, open circular (oc) or supercoiled (sc), is indicated. Error bars show the SEM among biological replicates (*n* = 3).

To identify genes that play a role in the production of a rDNA-dependent senescence signal, we designed a screening system based on the finding that overexpression of *FOB1* decreases replicative life span (22). The overexpression of *FOB1* seems to be stressful and harmful which could be due to an increased amount of senescence signal. We first confirmed the relationship between the amount of Fob1 and replicative life span (Fig. 2A and B). The *FOB1* gene (including its own promoter) was cloned into YCp and YEp vectors (38), which are single and multi-copy plasmids in a yeast cell, respectively. As shown in Fig. 2A, Fob1 protein-levels increased in the yeast strains along with the expected increase in copy number of *FOB1* (*FOB1p*): production of Fob1 in strains expressing the protein from the high-copy YEp-*FOB1* plasmid was about 10-fold higher than from YCp-*FOB1*. Overproduction of Fob1 also has a negative effect on replicative life span, which we found when monitoring the number of cell divisions of mother cells by counting these under a microscope (39). As shown in Fig. 2B, the replicative life span in cells with YEp-*FOB1* (ave. 11.3) was much shorter than that of cells only carrying the vector (ave. 19.9). These results confirmed that replicative life span decreases when Fob1 is overproduced.

We next tested the effect of overexpressing *FOB1* on the rDNA stability by pulsed-field gel electrophoresis in which unstable rDNAs are observed as smeared bands of chromosome XII due to copy number variation (4). The smearing of the bands for chromosome XII in YEp-*FOB1* strains pointed to unstable rDNA (Fig. 2C) (29). Furthermore, ERCs, products of rDNA recombination, were also increased in *FOB1*-overexpressing strains (Fig. 2D). These results suggested that a further increase in the levels of Fob1 might have a severe negative effect on the ability of strains to grow and form colonies. This possibility inspired us to design a screen to isolate mutants that could grow normally under conditions when Fob1 levels become growth-inhibitory.

**FIG 3:**
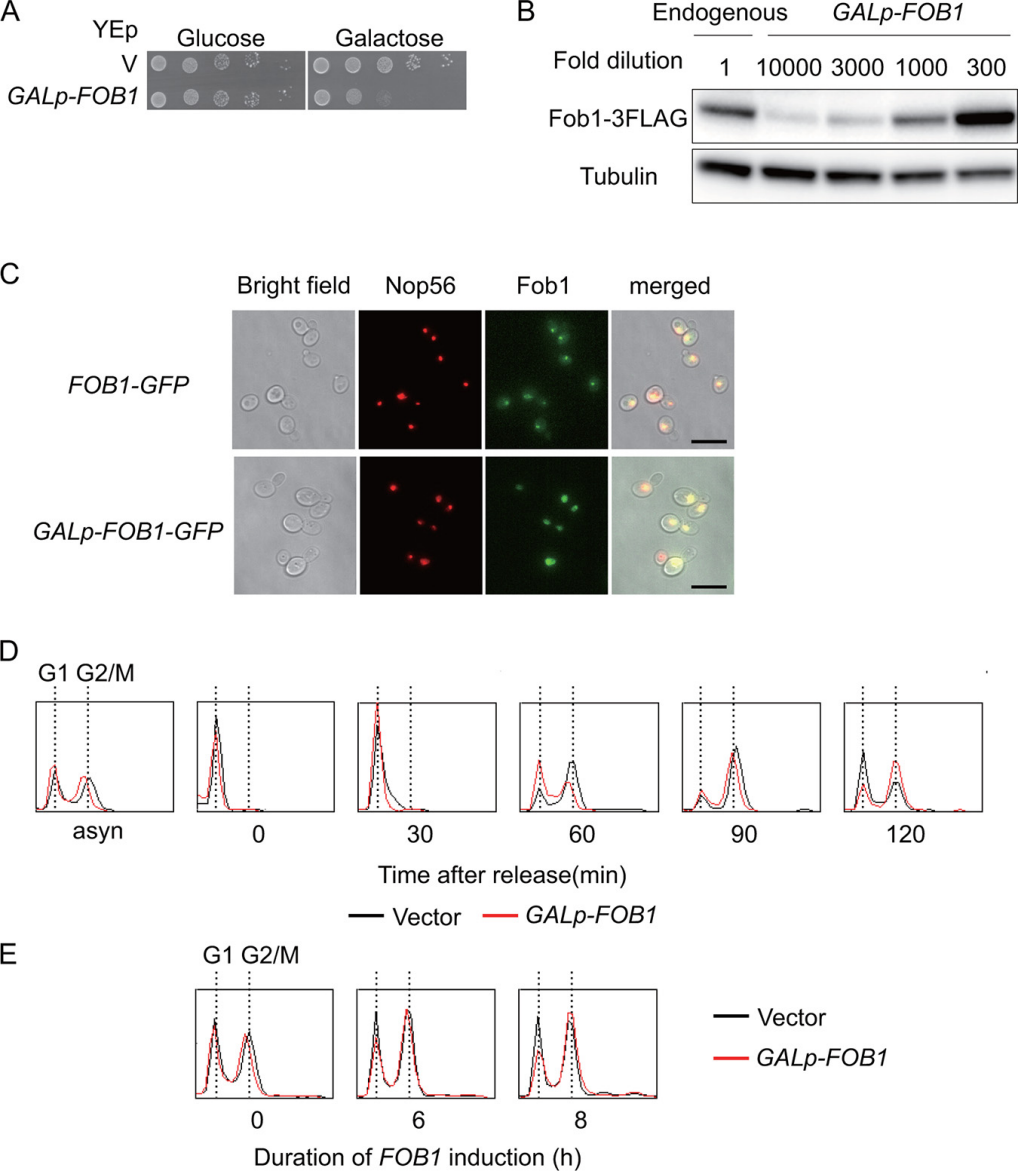
Characterization of *FOB1* overexpression from galactose inducible promoter (∼500-fold increase). (A) Serial dilution growth assay. Cells were harboring a multi-copy plasmid (V) with or without *FOB1* under the control of the *GAL7* promoter (*GALp-FOB1)*. Serial dilutions of cells cultured under non-inducing conditions were spotted on selective media repressing (glucose) or inducing (galactose) *FOB1* expression. (B) Estimation of Fob1 levels due to overexpression. Strains with endogenously FLAG-tagged *FOB1* (endogenous) and *GALp-FOB1* were analyzed by Western blotting after 6 h galactose induction using anti-FLAG and anti-tubulin antibodies. The protein samples had been diluted as indicated. For details see Materials and Methods. (C) Fluorescence microscopy localizing Fob1 in yeast cells. GFP-tagged Fob1 was expressed from the endogenous gene (*FOB1-GFP*) or from the gene placed under the control of a galactose inducible promoter on a multi-copy plasmid (*GALp-FOB1-GFP*). mCherry-tagged Nop56 was used as a control for nucleolar localization. The exposure times were 1 ms for *GALp-FOB1-GFP* (2nd right, bottom “Fob1”) and 200 ms for the other signals. (D) Analysis of DNA content by flow cytometry analysis. Cells growing asynchronously on raffinose (asyn) were arrested with alpha factor before galactose was added. DNA from cells collected at the indicated time points after release from the alpha factor, was analyzed as described in Materials and Methods. Traces for cells carrying the vector (black) or *GALp-FOB1* (red) peak when one genome complement, indicative for G1, is present and when replication has been completed and mitosis takes place (G2/M). (E) Flow cytometry analysis of asynchronous cells after addition of galactose. The duration of *FOB1* induction is indicated.

To establish a yeast strain in which *FOB1* expression could be maximized in a controllable manner, we placed the gene on the multi-copy plasmid under the control of a strong, galactose inducible promoter: The *FOB1-*gene carrying a C-terminal triple-FLAG (3FLAG) epitope tag was fused to the *GAL7* promoter, and this cassette was cloned into the YEp multi-copy vector, yielding YEp-*GALp-FOB1-3FLAG* (*GALp-FOB1*). To assess the effect of overexpressed Fob1 on cell growth, serial dilutions of cells transformed with YEp plasmids that had been cultured in non-inducible medium with raffinose as the carbon source, were spotted on plates with glucose, that represses the *GAL7* promoter, or galactose, that induces this promoter (Fig. 3A). In contrast to cells plated on glucose or control cells with empty vector, cells with *GALp-FOB1* showed poor growth on galactose (Fig. 3A). As shown by Western blotting, compared to the endogenous FLAG-tagged gene, expression from *GALp-FOB1* led to about 500-fold increase in the levels of Fob1 after normalization to tubulin (Fig. 3B). Like the chromosomally encoded Fob1-GFP (20, 40), Fob1-GFP expressed from *GALp-FOB1* localized to the nucleolus, as indicated by co-localization with the nucleolar marker Nop56 (Fig. 3C). We also monitored the effect of *FOB1* overexpression on the cell cycle by flow cytometry. As shown in Fig. 3D, *GALp-FOB1* cells were delayed at the release from G1 to S phase and during S-phase progression. Moreover, prolonged overexpression of Fob1 increased the fraction of cells in the G2/M phase (Fig. 3E). As elongation of the cell cycle (in both G1 and G2/M) is one of the phenotypes observed in yeast replicative aging (17–19), we speculate that too much senescence signal caused the delay.

### Screening of mutants suppressing *FOB1* overexpression effects.

**FIG 4:**
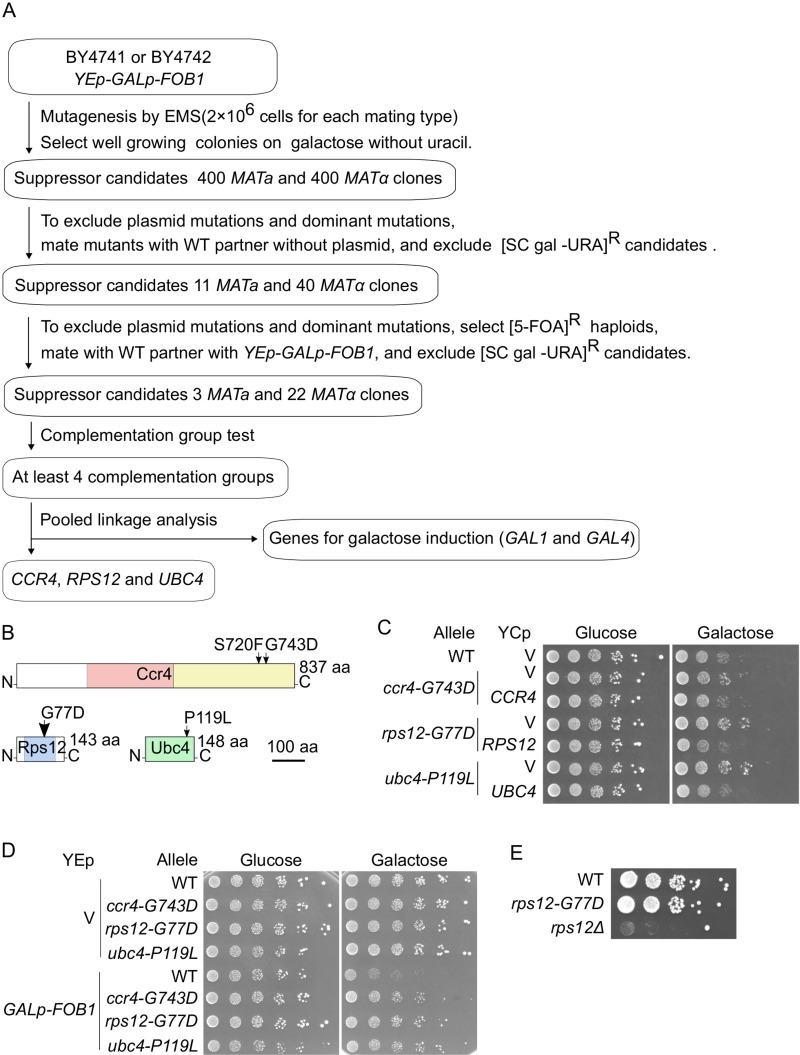
Isolation of mutants suppressing defects caused by *FOB1* overexpression. (A) Overview of the screen for suppressors of the growth inhibition caused by *FOB1* overexpression. See main text and Materials and Methods for details. (B) Schematic overview of suppressor proteins Ccr4, Rps12 and Ubc4 with mutations indicated (arrows) and conserved protein domains colored: leucine-rich repeat (COG5239; pink), and mRNA deadenylase (COG4886, yellow), ribosomal protein L7Ae/L30e/S12e/Gadd45 family (pfam01248; blue), UBCs super family (cl00154; green). (C) Serial dilution growth assay for suppressors harboring a YCp plasmid with the indicated wild-type allele or without (V). Cells cultured under non-inducing conditions were spotted on selective media repressing (glucose) or inducing (galactose) *FOB1* expression. (D) Serial dilution growth assay for wild type (WT) and suppressor mutants (*ccr4-G743D*, *rps12-G77D*, *ubc4-P119L*) harboring a YEp vector (V) or the YEp *GALp-FOB1* plasmid. Cells were cultured and spotted as in Fig. 3A. (E) Serial dilution growth assay for *rps12-G77D* and *rps12Δ.* Cells were spotted on YPD plates.

The severe growth defect caused by *FOB1* overexpression allowed us to conduct genetic screening of suppressor mutants that are expected to have defects in aging signaling. The details are described in Materials and Methods and the workflow is shown in Fig. 4A In short, we mutagenized *GALp-FOB1* cells by EMS and selected well growing colonies on a galactose plate. To exclude plasmid mutations and/or dominant mutants that cannot be classified by subsequent complementation tests, candidates were mated to wild-type partners without a plasmid. Resultant diploids with a suppressor phenotype harbored mutated plasmid and/or carried dominant mutations; these candidates were discarded. The remaining candidates were further tested to be recessive and dependent on chromosomal mutations. Plasmid-free mutants, selected by growth on media with 5FOA, were mated to wild-type partners harboring intact *GALp-FOB1* plasmid and selected for wild-type sensitivity to *FOB1* overexpression. Candidates with such a phenotype were carrying a recessive allele and classified into at least four complementation groups (Fig. 4A). Whole-genome sequences of representative candidates from each complementation group were analyzed with the mutation discovery pipeline Mudi to determine causative mutations (41).

As a result of the suppressor screen, we identified causative mutations in three genes: *CCR4* (S720F, and G743D), *RPS12* (G77D), and *UBC4* (P119L) (Fig. 4B). Complementation assays with YCp plasmids carrying wild-type alleles of the suppressor genes demonstrated that the identified alleles had been responsible for the suppression phenotype (Fig. 4C). Furthermore, reintroduction of each mutation into the wild-type strain suppressed the growth defect by *FOB1* overexpression: Compared with wild type, growth-recovery of strains carrying these mutations was only observed in *GALp-FOB1* cells plated on galactose medium. These results demonstrate that the suppressor effect of these mutations is tightly linked to overexpression of *FOB1* (Fig. 4D).

Ccr4 is a component of the CCR4-NOT deadenylase complex that is conserved in all eukaryotes, and this complex is involved in several cellular processes important for survival, such as transcription initiation, transcriptional elongation, and mRNA degradation (42–44). Mutations in this complex can lead to rDNA instability by accumulation of E-pro transcripts (37). The two alleles obtained in this screening were both located in the nuclease domain and close to catalytic site residues, mutation of which led to loss of deadenylase activity (D713A and D780A) (44).

Rps12 is a component of the small subunit of eukaryotic ribosomes (45). Most of the genes coding ribosomal proteins in yeast are essential and cannot be deleted unless paralogues with the same functions are available. Deletion of *RPS12* causes defects in growth, pre-rRNA processing and polysome content (46); the mutant, however, is viable although no *RPS12* paralogue exists. In contrast to the deletion mutant, growth of the *rps12-G77D* mutant we isolated is not affected by the mutation (Fig. 4E). Interestingly, deletion of *RPS12* is reported to extend replicative life span (31).

The *UBC4* gene-product is an E2 ubiquitin-conjugating enzyme that receives ubiquitin from an E1 ubiquitin-activating enzyme and transfers this to target proteins selected by an E3 ubiquitin ligase. Ubiquitination is well known as a marker for degradation of the modified protein by the proteasome, but it also functions in various cellular processes such as signals for cell cycle control, protein trafficking, and epigenetic regulation (47, 48). *UBC4* has a paralogue, *UBC5*, and is involved in the regulation of the cell cycle via APC, that can target Fob1, and helps to control other processes through interaction with a variety of E3 ligases (22, 33, 34).

### Alleles *rps12-G77D* and *ubc4-P119L* act in distinct pathways.

**FIG 5:**
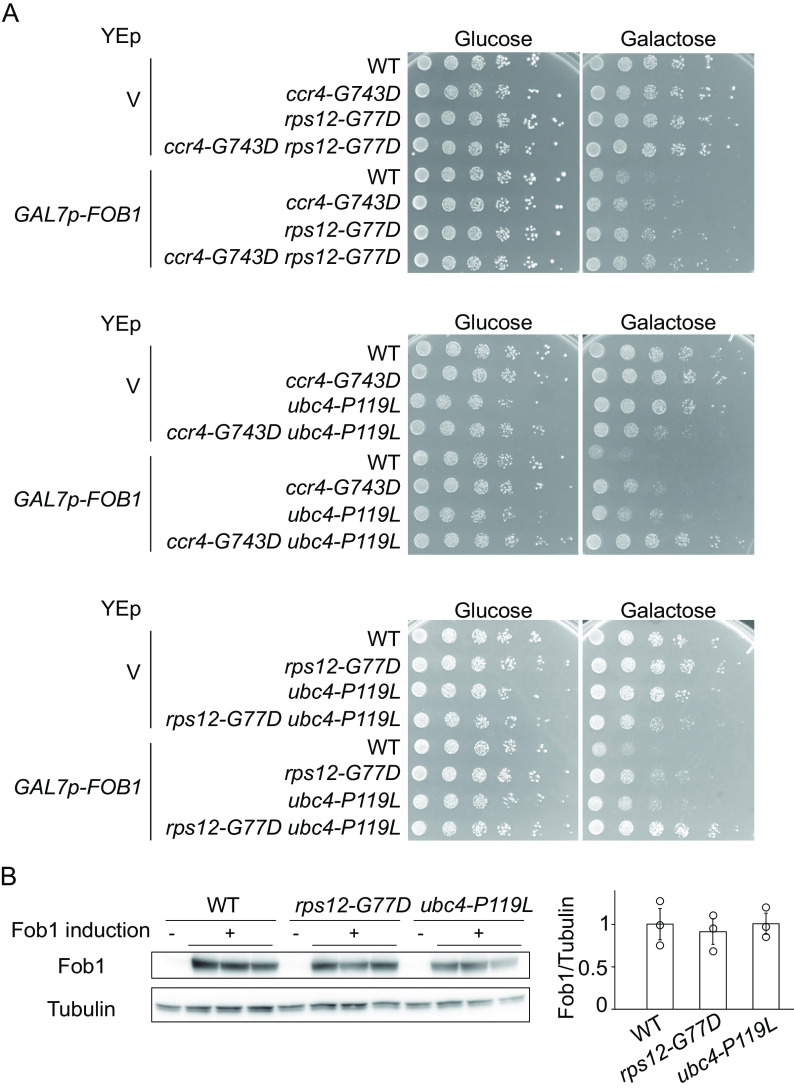
Genetic interaction of suppressor mutants. (A) Serial dilution growth assay for single and double mutants of growth-defect suppressors. Cells were spotted as in Fig. 3A. (B) Analysis of Fob1 levels in suppressor strains. FLAG-tagged Fob1 in wild type (WT), *rps12-G77D* and *ubc4-P119L* cells carrying *GALp-FOB1* was detected by Western blotting analysis (left panel) and quantified (panel to the right) as described in Materials and Methods. Error bars show SEM for biological replicates (*n* = 3).

Since the isolated mutations only partially suppressed growth defects by *FOB1* overexpression (Fig. 4D), the genes might function in distinct pathways. To determine whether these mutations suppress the growth inhibition caused by *FOB1* overexpression in a concerted manner or independently, double mutants were created and their growth on galactose was monitored (Fig. 5A). The *ccr4-G743D rps12-G77D* double mutant showed the same level of suppression as the *rps12-G77D* single mutant, suggesting that *ccr4-G743D* and *rps12-G77D* act in the same pathway (Fig. 5A). In contrast, compared to the single mutants, combination of alleles *ccr4-G743D* and *ubc4-P119L* or *rps12-G77D* and *ubc4-P119L*, suppressed growth on galactose medium better, indicating that the pathways involving *rps12-G77D* and *ubc4-P119L* or *ccr4-G743D* and *ubc4-P119L* are different (Fig. 5A).

In the screen, genes related to galactose induction, *GAL1* and *GAL4*, were also identified as carrying suppressor mutations (Fig. 4A). Gal4 is a transcriptional activator regulating *GAL*-gene promoters and becomes activated on galactose. It is therefore conceivable that the pathways of *rps12-G77D* and *ubc4-P119L* might suppress the growth defect through reduction of *GAL7p* activity and thereby the extent of *FOB1* overexpression. However, in cells transformed with *GALp-FOB1* and cultured in galactose media, Fob1 levels in suppressors *rps12-G77D* and *ubc4-P119L* were comparable to wild type, confirming that suppression by these alleles is not mediated by a decrease of Fob1 accumulation (Fig. 5B).

### Replicative life span was slightly extended in the *rps12* and *ubc4* but not in *ccr4* mutants.

**FIG 6:**
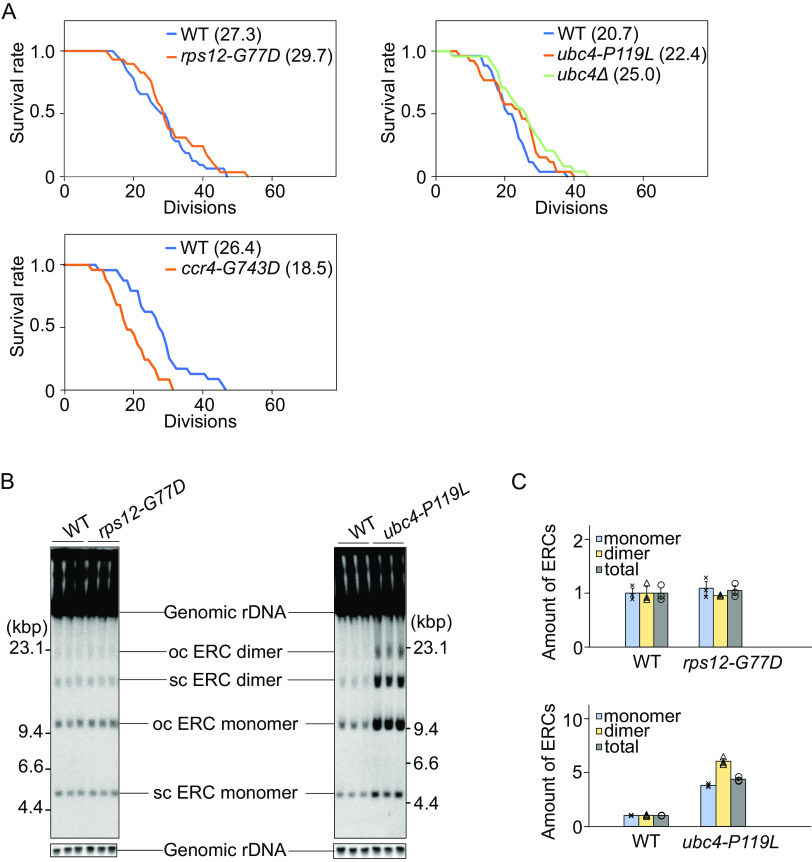
Replicative life span and ERC formation in suppressor mutants. (A) Survival curves for replicative life span of wild type (WT) and suppressors. The number of daughter cells that budded from each mother cell was counted. The number of mother cells counted, *n* = 32 (WT for *rps12)*, *29* (rps12-G77D), 26 (WT for *ubc4*), 26 (*ubc4-P119L*), 26 (WT for *ccr4*), and 25 (*ccr4*). Numbers in brackets indicate the mean replicative life span. (B) Analysis of extrachromosomal rDNA circles in suppressor strains. ERCs accumulating in the indicated strains were detected by Southern hybridization using a probe that covers the E-pro locus, as shown in Fig. 2D (ERC). The form of ERCs, open circular (oc) or supercoiled (sc), is indicated. (C) Signal intensities of ERCs per genomic rDNA normalized to wild type (WT). Error bars show SEM among biological replicates (*n* = 3).

If the isolated alleles had an impact on processes that generated a senescence signal, we would expect that replicative life span would be extended in these mutants without *FOB1* overexpression. When we measured replicative life span, the *rps12-G77D* and *ubc4-P119L* allele tended to extend this (Fig. 6A). As mentioned above, deletion of *RPS12* leads to a longer replicative life span than wild-type which was much greater than the increase in *rps12-G77D* (31). Contrary to *rps12Δ*, the growth of *rps12-G77D* was identical to that of wild type. These results suggest that *RPS12* affects replicative life span independent of growth. We also measured the replicative life span of a strain carrying a deletion of *UBC4* (*ubc4Δ*) and found that *ubc4Δ* also extended its replicative life span (Fig. 6A). In contrast, the replicative life span of *ccr4-G743D* was clearly shorter, suggesting that this allele is differently affecting processes that convey a senescence signal. In line with this finding, we have reported that in mutants of the CCR4/NOT complex high levels of ERCs are formed (37), which, as described in the introduction, can promote senescence in budding yeast. Because *ccr4-G743D* seems not directly related to the formation of a senescence signal, we decided to restrict further analyses to the *rps12-G77D* and *ubc4-P119L* mutants. Otherwise, since *CCR4* is involved in various aspects of cellular regulations (36), multifunction defects in the *ccr4-G743D* mutant might induce pleiotropic effects on distinct senescence signals and shorter replicative life span.

As the accumulation of ERCs can be indicative for increased rDNA instability and has been linked to reduced replicative life span (28) we measured the extent of ERC-formation in the *rps12-G77D* and *ubc4-P119L* mutants. Total DNA was isolated and analyzed by gel electrophoresis, as described in Materials and Methods. As shown in Fig. 6B and C, the amounts of ERC in the *rps12-G77D* mutant were comparable to those in the wild type. In contrast, in the *ubc4-P119L* mutant, ERCs accumulated to higher levels than in the wild type. These findings suggest that not ERC accumulation, but other pathways extend replicative life span in the *ubc4-P119L* mutant.

### Non-coding transcripts derived from E-pro drastically accumulated in *rps12-G77D*.

**FIG 7:**
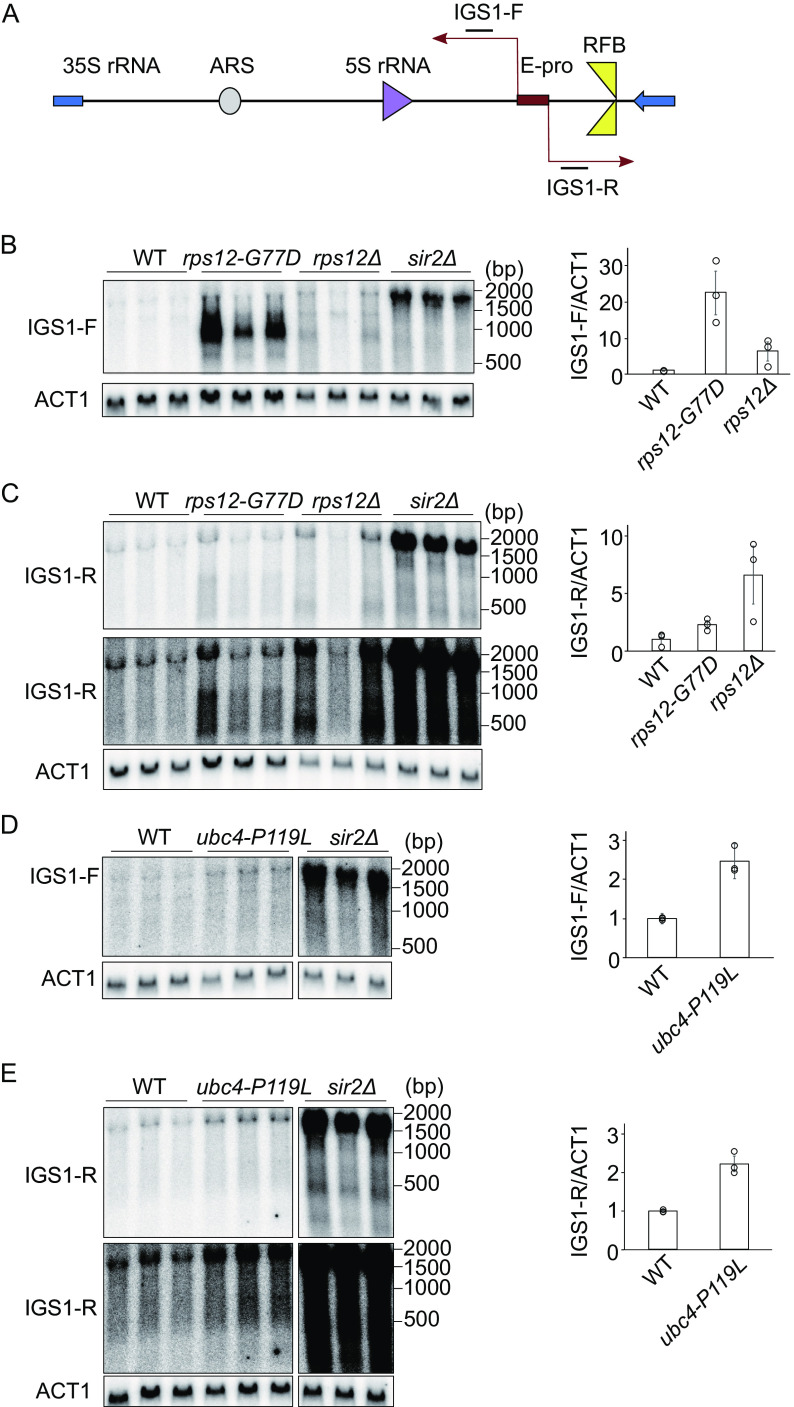
Intergenic spacer transcripts initiating at E-pro in suppressor mutants. (A) Schematic representation of the transcription from E-pro. (B-E) Total RNA isolated from the indicated strains was analyzed by Northern blotting as described in Materials and Methods. Blots were hybridized to probes for IGS1-F (B, D) or IGS1-R (C, E). As a loading control the mRNA for ACT1 was probed for. Signal intensities of IGS per ACT1 are shown in the right panel of each blot. The total signals for IGS in the area shown in B–E divided by ACT1 signal was normalized to that of wild type (WT). Error bars indicate the SEM among biological replicates (*n* = 3).

Active non-coding transcription from the E-pro is also a senescence promoting factor that shortens the replicative life span (5). For example, E-pro-driven transcription is significantly increased in the absence of Sir2 (Fig. 7). This interferes with the association of cohesin to the rDNA, which leads to enhanced rDNA instability that shortens the replicative life span (24). We checked the activity of the E-pro locus by probing for intergenic spacer transcripts (IGS) in the *rps12-G77D* and *ubc4-P119L* mutants. Transcripts produced from either strand (IGS1-F and IGS1-R) were detected by specific probes (Fig. 7A). In the *rps12-G77D* mutant, the amount of these transcripts increased compared to the wild type. Especially IGS1-F transcripts, co-directional with 35S transcription, were highly abundant (∼22 times to wild-type) and in comparable amounts to those in the *sir2* mutant though their length was much shorter (Fig. 7B). The short IGS1-F transcripts were also observed in the absence of the Rps12, although at quite a reduced level (∼6 times) (Fig. 7B, lane *rps12Δ*).

In contrast to IGS1-F, the IGS1-R transcripts (running in the opposite direction and co-directional with 5S transcription), were only slightly (∼2.8 times to wild-type) enhanced compared to the wild type. This was observed when *RPS12* was either mutated or absent (Fig. 7C and 5G).

In the *ubc4-P119L* mutant, E-pro derived transcripts increased compared to wild-type (∼2.5 time, IGS1-F; ∼2.3 times, IGS1-R), but not as much as in the *sir2Δ* or IGS1-F transcript in *rps12-G77D* (Fig. 7D). The increased amount of ERC in the *ubc4-P119L* mutant may explain a higher level of E-pro transcripts (Fig. 6B, 6C, 7D, and 7E).

E-pro transcripts have been found as senescence-promoting factors and were upregulated in both mutants, especially in *rps12-G77D*, still their replicative life span was extended (Fig. 6A). Therefore, either a senescence signal from ERCs has been interfered with in these mutants or a different process triggering senescence has been affected.

### DNA breaks are reduced in *rps12-G77D*.

Other putative senescence factors are replication fork arrest and DSB at the RFB site that trigger rDNA recombination (11–13). As mentioned in the Introduction section, we once established a less replication-initiation strain in which a part of rARS (ribosomal autonomously replicating sequence) is deleted (29). This rARS modified strain have more unstable rDNA (more recombination) and shorter replicative life span though they have less ERC (29) than the wild-type strain. These suggest that rDNA instability itself affects replicative life span. Therefore, we tested the fork arrest and DSB.

**FIG 8:**
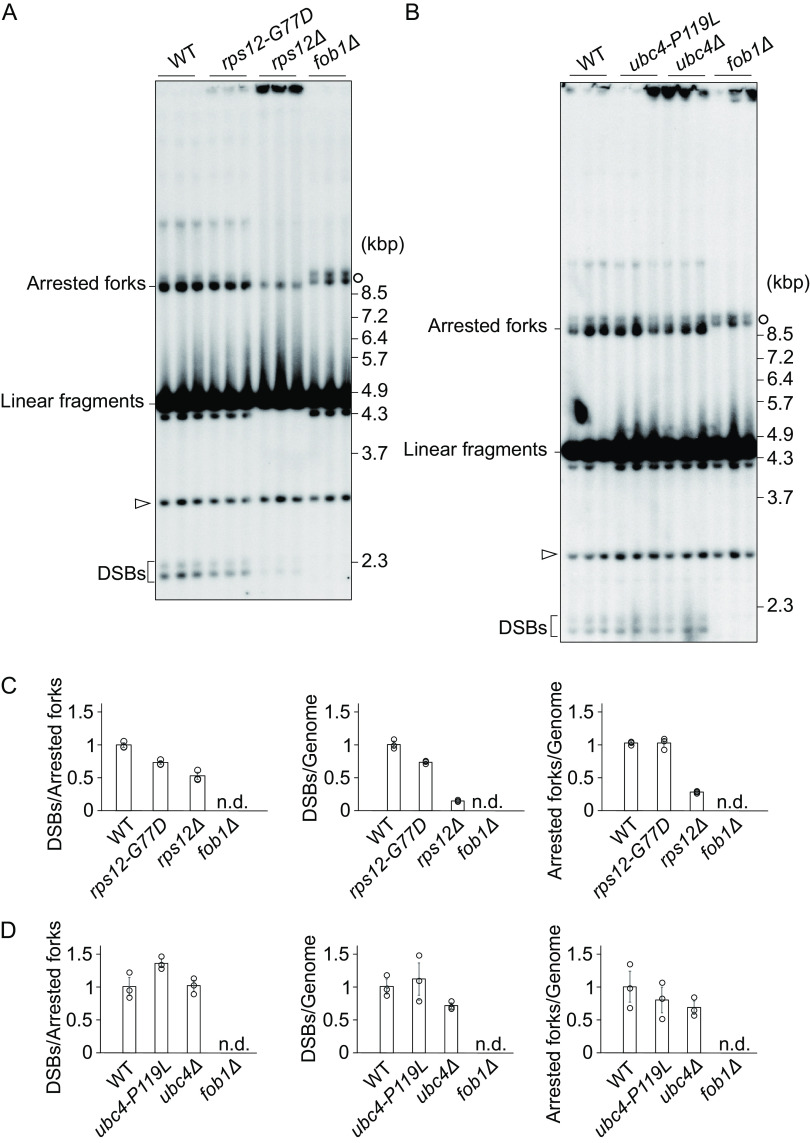
Fob1 dependent double strand breaks in rDNA of suppressor mutants. Genomic DNA of wild type (WT), *rps12* (A, C) or *ubc4* (B, D) strains, cut with BglII, was monitored by agarose gel electrophoresis. (A) and (B) A probe hybridizing nearby the ARS sequence and 35S promoter (see Fig. 1, DSB) was used to detect the indicated intermediates including terminal fragments containing the telomere-proximal rDNA repeat and its adjacent non-rDNA fragments (open arrows). In the absence of Fob1 (*fob1Δ*), intermediates with replication forks that just entered the 35S rRNA region are prevalent (open circles). (C) and (D) The signal intensity of DSBs per arrested fork, and of DSBs or arrested forks per genome was calculated. Error bars show the SEM among biological replicates (*n* = 3).

Genomic DNA was isolated from cells in the exponential growth phase, digested with BglII and separated by agarose gel electrophoresis to observe replication intermediates and double strand breaks. Southern blots of these gels were hybridized to a probe nearby rARS close to the 35S promoter (Fig. 1). We used the *fob1*Δ mutant as a negative control in which there is neither fork arrest nor DSB formation (11). The results are shown in Fig. 8 in which various intermediates are indicated. The frequency of fork arrest can be measured from the intensity of the band “Arrested forks” that corresponds to Y-shaped replication fork intermediates stalled at the RFB site. As for the frequency of DSBs at the RFB site, the small band near the bottom of the gel was measured (Fig. 8A and B). DSB bands intensities were quantified and normalized to total rDNA intensity or that of arrested forks (Fig. 8C and D). In the case of the *rps12-G77D* mutant, estimated number of arrested forks per genome were comparable to those of the wild-type strain (Fig. 8A and C, right), while the frequency of DSBs were reduced compared to the wild type (Fig. 8A and C, left and center). If DSBs are linked to senescence signal production, this observation supports the hypothesis that the *rps12-G77D* allele participates in the attenuation of this signal.

We also tried to establish this analysis for the *rps12* deletion strain and found that the frequency of DSBs also appeared to be lower than wild type (Fig. 8A and C, left and middle). This observation is consistent with a role for Rps12 in the onset of DSBs and in the formation of a senescence signal if this is connected to the frequency of DSBs. In the *rps12Δ* mutant, the ratio of arrested forks per genome was also lower than wild type (Fig. 8A and C, right). It is worth noting that in line with its slow growth (Fig. 4E), this deletion strain provided only a small percentage of cells in S-phase and a very limited number of arrested forks (Fig. 8A).

In the *ubc4-P119L* mutant, both the frequencies of arrested forks and DSBs were comparable to those determined for the wild-type strain (Fig. 8B and D). Overall, this analysis suggests that when the frequency of DSBs is related to senescence signal production in the *rps12* mutants, that such a signal is generated differently in the pathway involving *ubc4-P119L*.

## DISCUSSION

Genome instability is known to induce cellular senescence and to restrict life span (1). Several factors have been considered that lead to senescence in relation to genome instability, such as DSB, noncoding transcription, resection, single strand DNA, recombination events. Insufficient information, however, is available to determine which factor, if any, is the main trigger for senescence. In this study, we aimed to address this question and used Fob1, that associates with the fragile rDNA and plays a role in senescence, as a stressor. Overexpression of *FOB1* in the cell leads to a change in the cell cycle and causes phenotypes associated with onset of senescence: replicative life span reduction and high levels of ERC formation that are linked to variability in the rDNA locus (Fig. 2). We identified alleles of *CCR4*, *RPS12*, and *UBC4* as suppressors of a growth defect induced by *FOB1* overexpression. Of these, the *rps12-G77D* and *ubc4-P119L* alleles could be linked to onset of senescence.

ERC accumulation is a cause of senescence and finally restricts the replicative life span in budding yeast (28). By single cell imaging analysis, it is observed that ERC levels exponentially increase near the end of a life cycle (17). They seem to titrate factors for genome maintenance and cell growth, such as histones and cell cycle regulatory proteins (18, 19). In fact, supplementing factors to compensate expected shortages can extend replicative life span (18, 49). Here, our analysis of the *ubc4-P119L* mutant demonstrates that the amount of ERC can increase (Fig. 6B) while replicative life span is extended in both point and deletion mutants (Fig. 6A). One possible explanation is that Ubc4, as a ubiquitin-conjugating enzyme, is involved in the regulation of factors titrated by ERC, such as histones and cell cycle regulatory proteins, and in the mutant, these factors remain available. Actually, histone H3 is known to be a target of Ubc4 (50). Conversely, the efficient supply of factors for ERC maintenance and production may lead to ERC accumulation in the *ubc4-P119L* mutant.

The *UBC4* gene-product acts as the E2 enzyme for the anaphase promoting complex APC, which is an E3-ligase, and participates in the self-regulation of its catalytic core, subunit Apc11 (33). The APC complex controls the passage through M and G1 during the cell cycle by ubiquitin dependent targeting of proteins, one of which is Fob1: A subunit of the APC complex, Apc5, is able to physically interact with Fob1 (22). Although Apc5 is reported to degrade Fob1, in *ubc4-P119L* the amount of overexpressed Fob1 was comparable to that of wild type.

In the *rps12-G77D* mutant, the replicative life span appears slightly extended (Fig. 6A) while in the absence of the protein this is reported to be longer (31), despite a severe growth defect of the deletion mutant (Fig. 4E) (46). As Rps12 is a component of ribosomes, its absence can result in reduced protein synthesis and thereby lessens metabolic activities, leading to an extension of replicative life span. This is not specific for Rps12. Mutations of some other ribosomal protein genes are known to have a similar effect (31). The synthesis of ribosomes is the most energy consuming cellular process whereby ROS and other by-products are generated that have a negative impact on replicative life span. Reduced ribosome biogenesis, as caused by calorie restriction or a defective TOR pathway, is therefore expected to extend replicative life span, which is supported by evidence (51, 52).

The growth in the *rps12-G77D* mutant was normal, indicating that adequate numbers of functional ribosomes are synthesized. It was isolated as a suppressor of the growth-defect induced by *FOB1* overexpression that can be linked to rDNA instability and reduced replicative life span (Fig. 2B to D). This suggests that Rps12 has an additional function to being a component of ribosomes. In contrast to *SIR2*, despite a comparable increase of noncoding transcription from E-pro (Fig. 7B) (20) the replicative life span was not reduced in *rps12-G77D*. Interestingly, accumulation of IGS transcripts in this mutant was asymmetric; only those transcripts in the direction of 35S transcription and counter to that of the replication fork were very abundant, although shorter than those observed in the absence of Sir2 or after mutation of CCR4/NOT subunits (Fig. 7A) (37). Despite the accumulation of IGS1-F transcripts in the mutant, the ERC was not increased, indicating that unequal sister-chromatid recombination was not activated (Fig. 6B). Possibly, the asymmetric formation of shorter transcripts from E-pro might not be sufficient for dissociating cohesin from the rDNA to an extent that such recombination can occur. In view of genetic analysis pointing to the possibility that the *ccr4-G743D* and *rps12-G77D* alleles act in the same pathway (Fig. 5A), Ccr4 may be responsible for the shorter IGS1-F transcripts due to its RNA degradation activity (37).

Asymmetric formation of IGS1-F transcripts of reduced length could also lead to a decrease in the frequency of DSBs that trigger recombination events and are dependent on fork-arresting by Fob1/RFB. Compared with wild type cells, less DSBs are formed in the rDNA near the Fob1/RFB site in the *rps12-G77D* mutant. Maybe a combination of such events can prevent the level of DNA damage induced by Fob1 that reduces replicative life span. Further research has to address how a ribosomal protein allele can accomplish this, while in another route, alteration of a component involved in ubiquitination of Fob1 relieves the cell from the same stress.

## MATERIALS AND METHODS

### Yeast strains and culture conditions.

Yeast strains used in this study are listed the file. Yeast cells without plasmids were cultured in YPD media, that is YP (10 g/L yeast extract, 20 g/L peptone) with 20 g/L glucose (56). 20 g/L Difco Bacto Agar (BD bioscience) was added for plate medium. Synthetic complete media (SC) without uracil used in this study was modified from Hartwell’s complete media [1xYNB powder w/o AA (Thermo Fisher Scientific), 20 g/L carbon source (glucose, galactose or raffinose), 20 mg/L l-arginine HCl, 60 mg/L L-Tyrosine, 80 mg/L l-isoleucine, 50 mg/L l-phenylalanine, 100 mg/L l-glutamic acid, 100 mg/L L-aspartic acid, 150 mg/L l-valine, 200 mg/L l-threonine, 400 mg/L l-serine, 40 mg/L adenine sulfate, 60 mg/L l-leucine, 40 mg/L L-tryptophan, 20 mg/L l-histidine HCl, 20 mg/L l-methionine, 120 mg/L l-lysine HCl and 20 g/L uracil] (56). For the construction of deletion and point mutants, antibiotics were added to YPD plate media with concentrations of 200 μg/mL for G418 (Sigma-Aldrich), 200 μg/mL Hygromycin B (Wako), and 100 μg/mL clonNAT (Werner BioAgent). For 5-Fluoroortic acid (5-FOA, Sigma-Aldrich) selection, 1g/L 5-FOA was added to SC plate media.

### Yeast strain and plasmid constructions.

Strains used in this work are described in Table S1 and plasmids in Table S2 in the supplemental material. Oligonucleotides used in PCRs are in Table S3. PCRs were performed using KOD Fx Neo polymerase (Toyobo). Yeast cells transformed with plasmids were selected on SC glucose medium without uracil for strains harboring YCplac33 or YEplac195, or without uracil and leucine for strains harboring YEplac195 and YCplac111. When introducing fragments with *kanMX* or *hphMX*, transformed cells were cultured on YPD plates overnight and the cells were replicated to YPD plates containing G418 (Sigma-Aldrich) or Hygromycin B (Wako) and cultured for several days.

Deletion-mutants for *FOB1* and *SIR2* were constructed as follows: DNA fragments *fob1Δ::hphMX* and *sir2Δ::hphMX* were amplified from pFA6A-hphMX with sy86/sy87 and MS75/MS76 primer pairs and transformed into yeast strain SY1. To delete *RPS12*, DNA fragment *rps12Δ::kanMX* was amplified from pFA6A-kanMX with sy69/sy70 and transformed into SY3. The resultant diploid *rps12Δ::kanMX/RPS12* were sporulated and haploid *rps12Δ::kanMX* was obtained by tetrad dissection.

YEplac195-FOB1pFOB1 was generated by recombination of YEplac195 (Gietz and Sugino, 1988) with the FOB1pFOB1 fragment from YCplac33-FOB1pFOB1 (Iida et al. 2019). YCplac33 (YCp; Gietz and Sugino, 1988), YCplac33-FOB1pFOB1 (YCp-*FOB1*), YEplac195 (YEp) and YEplac195-FOB1pFOB1 (YEp-*FOB1*) were transformed into yeast strain NOY408-1b for analyses in Fig. 2.

The *GAL7p-FOB1* fragment was amplified from the yeast strain YTT28 and cloned into YEplac195 to generate YEplac195-GAL7p-FOB1. YEplac195-GAL7p-FOB1-3FLAG (*GALp-FOB1*) was constructed by 3FLAG tagging to the *FOB1* gene on YEplac195-GAL7p-FOB1.

*GALp-FOB1* was transformed into SY1, SY268, SY683 and SY705 for analyses in Fig. 3 and 4.

To construct endogenously FLAG-tagged *FOB1*, the fragment *FOB1-3FLAG-ADH1ter*-*URA3* was amplified by fusing the following PCR fragments with sy40/sy38: *FOB1*-3FLAG amplified from YEplac195-GAL7p-FOB1-3FLAG with sy40/sy121; the *ADH1* terminator amplified from pKT129 with sy123/sy124; *URA3* amplified from YIplac211 with sy109/sy110; and the *FOB1* terminator amplified from the wild-type genome with sy124/sy38. This fragment was transformed to yeast strain SY1, yielding SY299.

To observe Fob1 localization by fluorescence microscope, YEplac195-GAL7p-FOB1-GFP (*GALpFOB1-GFP*) was constructed by replacing the FLAG3-tag in *GALpFOB1* with a fragment amplified from pKT127 using sy183/sy184. To construct endogenously GFP-tagged *FOB1*, a *FOB1-GFP* fragment was amplified from *GALpFOB1-GFP* with sy40/sy123 and fused with *URA3-FOB1ter* amplified from SY299 with sy124/sy38. The fusion PCR was done with sy38/sy40 and the product was integrated into SY684. Strain SY684 was also the recipient for *GALpFOB1-GFP*.

Since Fob1 is reported to localize to the nucleolus (20, 40), endogenous *NOP56*, a nucleolar marker, was tagged with mCherry by integrating a fragment amplified from the mCherry-HIS3MX plasmid with oli764/oli765 which was transformed into YTT121.

To re-construct suppressor strains and to test combinations of suppressor alleles, coding and terminator fragments of suppressor alleles cloned in YIplac211 (see below) were amplified and fused to *kanMX* or *hphMX* amplified with sy103/sy104 from pFA6a-MN3HA-kanMX or pFA6a-MN3HA-hphMX (26). Primers used for the fusion PCRs were as follows: sy140, sy149, sy154, and sy178 for *ccr4*; sy115, sy116, sy117, and sy118 for *rps12*; sy101, sy102, sy105, and sy106 for *ubc4*. The resultant fragments were transformed into yeast strains SY1, SY268, SY683, and SY705.

For complementation analyses, the wild-type and suppressor mutation alleles of *CCR4*, *RPS12* and *UBC4* were amplified by PCR from genomic DNA using primer combinations sy128/sy129, sy57/sy58, and sy61/sy62, respectively. The amplified fragments were cloned into YIplac211 and YCplac111 (38). *GALp-FOB1* and YCplac111 or YCplac111 with the cloned alleles were transformed into each re-constructed suppressor and used for Fig. 4.

### Serial dilution growth assays.

Cells grown on SC glucose without uracil plates were inoculated into 3 mL of SC raffinose medium without uracil and cultured at 30°C. Fully grown cells were washed in water and adjusted to 2 × 106 cells/mL. Then, 5 μL of serially, 5-fold diluted cell suspensions were spotted on SC glucose or galactose without uracil and incubated at 30°C for 3 to 4 d.

### Replicative life span analysis.

Replicative life span analysis was performed as previously described (39) using a dissection microscope MSM 400 (Singer). Cells without buds were isolated on plates and their division was tracked. While the larger mother cell was removed, cells grown from the first buds were kept and the number of divisions these cells could support were counted. After each cell-division, cells derived from a newly formed bud were removed. Cells with plasmid were cultured on SC glucose without uracil (Fig. 2B) and cells without plasmid were cultured on YPD plates (Fig. 6A). Cells were cultured at 30°C during the daytime and were kept at 8°C overnight.

### Fluorescence Activated Cell Sorter (FACS) analysis.

To prepare synchronous cultures for FACS analysis, fully grown cells cultured in SC glucose media without uracil were inoculated in SC raffinose media without uracil and were cultured until they reached a density of about 1 × 10^7^ cells (2∼ doublings after inoculation). The cells were once collected and washed with YPR media (YP with 20 g/L raffinose), then suspended in YPR media containing 4 μM α-factor at a cell density of 6 × 10^6^ cells. Cells were incubated at 30°C for 1.5 h and, after addition of 200 g/L galactose to a final concentration of 20 g/L to induce *FOB1* expression, further incubated at 30°C for 1.5 h. To release cells from the α-factor block, cells were collected by centrifugation, washed twice with YPGal media (YP with 20 g/L galactose), and resuspended at ∼1.5 × 10^7^ cells/mL in YPGal media and incubated at 30°C. After the release of α-factor, cells were harvested at several time points, collected by centrifugation, resuspended in 70% vol/vol ethanol, and stored at –20°C. For the flow cytometry analysis of cells exposed to prolonged *FOB1* induction (Fig. 3E), galactose was added to asynchronously growing cells and samples were harvested after 6 and 8 h.

Flow cytometry analysis was basically performed as previously described (53). The procedures were as follows: cells stored in 70% ethanol were centrifuged, and ethanol was removed. The cells were resuspended in 200 μL of 50 mM sodium citrate (pH 7.4) containing 0.25 mg/mL RNaseA (Macherey-Nagel) and incubated at 37°C for 1 h. After RNaseA treatment, 100 μL 50 mM sodium citrate (pH 7.4) containing 0.5 mg/mL Proteinase K (Nacalai) was added, and cells were incubated at 50°C for 1 h. Finally, 300 μL of 50 mM sodium citrate (pH 7.4) containing 4 μg/mL propidium iodide (Sigma-Aldrich) were added and stored at 4°C. Cells were sonicated and diluted with 50 mM sodium citrate (pH 7.4) containing 2 μg/mL propidium iodide if necessary. For Flow cytometry analysis BD Accuri C6 Flow Cytometer (BD Bioscience) was used.

### Fluorescent microscopy.

Fluorescence microscopy was performed as previously described (54) using an ECLIPSE Ti microscope (Nikon) fitted with a Zyla 4.2P sCMOS (Andor Technology) camera. Cells were cultured in SC raffinose without uracil to the exponential growth phase and *FOB1* expression was induced by adding 20 g/L galactose to a concentration of 2 g/L. After 6 h of growth in the presence of galactose, cells were harvested, fixed with paraformaldehyde and washed with 1x PBS. Fluorescence signals were captured with a through-focus stack of 11 0.3 μm steps and was measured by ImageJ (Fiji). Z-stacks of the max intensity are shown in the figures.

### Screen for suppressors of Fob1-induced growth inhibition.

Mutagenesis was basically performed as previously described (26). Cells of both mating types for SY121 (BY4741 with YEp) and SY127 (BY4742 with *GALp-FOB1*) (Table S1 in the supplemental material), fully grown in SC raffinose media without uracil, were inoculated in SC galactose media without uracil and incubated for 4 h to induce *FOB1* expression. For mutagenesis, 2 × 10^6^ cells were suspended in 90 μL sterilized water and 100 μL PBS and 10 μL EMS (ethyl methanesulfonate) were added. The mix was incubated at 30°C for 20 min after which the reaction was stopped by addition of 800 μL 12.5% sodium thiosulfate. The cells were spread on SC galactose without uracil at a density of 1 × 10^5^ cells per plate and incubated at 30°C for 3 days. At this time point, for each mating type 320 colonies were isolated as suppressors (under these conditions, wild-type cells over-expressing *FOB1* would not have formed colonies large enough to be picked) and re-streaked on fresh SC galactose plates without uracil. Additionally, 4 days from EMS treatment, 94 more colonies were picked for each mating type and re-streaked.

The isolated suppressors were supposed to include strains that carried mutations in the plasmid that would decrease *FOB1* expression, and dominant chromosomal mutations, which cannot be classified by complementation tests. To exclude strains with such mutations, candidates consistently growing on galactose were mated to the wild-type parental strain (not harboring a plasmid). The resultant diploids were diluted in SC raffinose without uracil and spotted on SC galactose plates without uracil. If a mutation was recessive and genomic, the diploid would show the wild-type like growth defect on galactose. If the mutation was dominant or linked to the plasmid, however, the diploid would grow on galactose like the suppressor.

To further confirm that plasmid mutations and dominant mutations had been excluded, haploid candidates with putative recessive chromosomal mutations, were cultured in YPD and spotted on SC glucose plates with 5-FOA, selecting for *ura3*- phenotypes. 5FOA-resistant cells, that had dropped their plasmid, were mated to their parental strains harboring intact *GALp-FOB1*. Diploids that could grow on SC galactose plates without uracil were indicative for causative mutations that were on plasmid or dominant, and those candidates were excluded.

Recessive suppressors were classified by complementation tests in which *MATa* and *MATa* mutants were crossed to each other and plated on SC galactose without uracil.

Alleles with mutations were isolated by tetrad dissection, and mutations were identified by next generation sequencing. For this, 10 μg of genomic DNA in 100 μL of TE buffer (pH 8.0) extracted from fully grown yeast cells was sheared at 4°C using a Covaris S220 ultrasonicator (Covaris) and a microtube AFA fiber (Covaris) with the following settings: peak power 140, duty factor 10, cycle/burst 200, duration 45 s. The shearing was confirmed by agarose gel electrophoresis, and DNA fragments from 300 to 500 bp were gel purified with a NucleoSpin Gel and PCR Clean-up kit (Macherey-Nagel). After end repair and A-tailing of 100 ng of extracted DNA using a HyperPrep Kit (KAPA biosystems), the treated DNA was purified with 1 vol of AMpureXP (Beckman Coulter) beads. With the purified genomic DNA fragments as templates, DNA libraries for NGS sequencing were prepared using TruSeqHT (Illumina) compatible adaptors and KAPA HiFi HotStart ReadyMix with the following PCR settings: 98°C 45 s, and then 6 cycles of 98°C 15 s, 60°C 30 s, 72°C 40 s. The amplified fragments were purified using 1 vol of AMpureXP (Beckman Coulter) beads. Equimolar mixtures of the libraries were sequenced on a MiSeq (Illumina) using 200 bp paired-end runs and the MiSeq reagent kit v3 (Illumina, MS-102-3003).

Causative mutations were identified by analyzing FASTQ files with the webtool Mudi (version2, http://naoii.nig.ac.jp/mudi_top.html) (41). Segregants conferring galactose-induced Fob1-suppression and obtained by tetrad dissection, would have causative mutations while other, non-causative mutations would be randomly distributed in mutant and wild-type like segregants. Causative mutations, marked by a high coverage, were identified by sequence alignment. Each identified gene was confirmed to be a suppressor by introducing a YCp plasmid harboring the wild-type gene and subsequent loss of the suppressor phenotype (as shown in Fig. 2C). Mutant alleles were then isolated by PCR and re-cloned (see above).

Gene domains were assessed using the NCBI Conserved Domain Database search facility at https://www.ncbi.nlm.nih.gov/Structure/cdd/wrpsb.cgi

### Western blot analysis.

For *FOB1* expression experiments using YCp-*FOB1* and YEp-*FOB1* (Fig. 2A), yeast cells in the exponential growth phase were used that had been cultured in SC glucose media without uracil to a cell density of 1 × 10^7^ cells/mL. For *FOB1* overexpression with *GALp-FOB1* (Fig. 3B and 5B), yeast cells in the exponential growth phase grown in SC raffinose medium without uracil to a cell density of about 3 × 10^6^ cells/mL were collected and samples reserved as a no *FOB1* overexpression control (-). To the remainder of these cells 20% galactose was added to a final concentration of 2% and the *FOB1* overexpression samples were collected after 6 h of incubation. Collected cells, washed once with ice cold water, were resuspended in 325 μL 430 mM NaOH, 0.68% vol/vol 2-mercaptoethanol and put on ice. After 15 min, 75 μL of 50% vol/vol trichloroacetate was added and incubation on ice continued for another 15 min. Cells collected by centrifugation were resuspended in 0.25 M Tris-HCl pH 6.8, 8% wt/vol SDS, 0.1% wt/vol bromophenol blue, 40% vol/vol glycerol, 0.1 M dithiothreitol and the extracts heat denatured at 65°C for 10 min. SDS/PAGE of 5 μL of protein sample was on 7.5% e-PAGEL (Atto) in 25 mM Tris, 192 mM Glycyne, 0.1% SDS at 10∼20 mA per gel. Proteins were transferred to immobilon-P PVDF membrane (Merck-Millipore) in 25 mM Tris, 192 mM glycine, 10% vol/vol methanol at 100 V for 1 h 4°C using a mini-transblot cell (Bio-Rad).

After transfer, the membrane was cut between the 75 kDa and 50 kDa marker bands that comigrated with Fob1 and α-tubulin, respectively, and blocked in PBS-T (1xPBS, 0.05% Tween20), 5% Skim milk (Wako) at room temperature for 1 h. For Fob1, Fob1-3FLAG and α-tubulin detection, membranes were incubated in blocking buffers containing anti-Fob1 (Santa Cruz, diluted 3 × 10^3^ fold), M2-HRP (Sigma-Aldrich, 2.5 × 10^5^ dilution) or anti-Tubulin-HRP (10 × 10^3^ dilution) at 4°C overnight. The membranes were washed three times 5 min with PBS-T at room temperature and, for Fob1 detection, incubated in blocking buffer with anti-rabbit IgG-HRP (5 × 10^3^ dilution) at room temperature for 1 h and washed. Signals, induced with Immobilon Western Chemiluminescent HRP Substrate (Merck Millipore), were captured with a Fusion FL4 system (Vilber Lourmat). Signal profiles of each lane were obtained, and the amount of each protein was defined as the area of each peak.

### Northern blot analysis.

Cells in the exponential growth phase grown in YPD media to a cell density of ∼2 × 10^7^ cells/mL were washed with ice cold water, resuspended in 200 μL of 10 mM Tris-HCl pH 7.5, 10 mM EDTA-Na pH 7.5, 0.5% wt/vol SDS) and 200 μL of phenol equilibrated with 50 mM sodium acetate (pH 5.2), and incubated at 65°C for 1 h. The suspension was centrifuged and the water-soluble fraction was extracted with 1 vol chloroform/phenol equilibrated with 50 mM sodium acetate (pH 5.2). The RNA was precipitated from the water layer (∼300 μL) with 40 μL 3 M sodium acetate and 1 mL 100% ethanol at –80°C overnight. RNA pellets were washed with ice cold 70% ethanol and dissolved in autoclaved, DEPC-treated water. RNA concentrations were measured by a NanoDrop ND-1000 spectrophotometer (Thermo Fisher Scientific).

For gel-electrophoresis, 20 μg RNA was denatured at 65°C for 10 min in a 20 μL mix with 8 μL formamide (Wako), 4 μL formaldehyde (Wako), 2 μL ethidium bromide, and 2 μL 10x MOPS buffer. After loading dye was added, the RNA samples were separated over 1% agarose gels containing 1x MOPS buffer and 6.2% formaldehyde. Electrophoresis was in 1x MOPS buffer at room temperature, initially at 25V for 20 min, then at 135V for 80 min.

After electrophoresis and washing the gel in DEPC-treated water, RNA separation was checked on a Fusion FL4 system (Vilber Lourmat). The gel was rinsed with 10 × SSC and the RNA was capillary-transferred to Hybond-N+ (GE Healthcare) using 10 × SSC overnight. After the transfer, the RNA was fixed to the membrane with 120,000 μJ/cm^2^ in a Stratalinker 1800 (Stratagene), washed in 5 × SSC and air-dried.

Hybridization was performed basically as described previously (37) in 15 mL of ULTRAhyb ultrasensitive hybridization buffer (Thermo Fisher Scientific). Random-primed probes were generated with 10 ng gel-purified DNA, amplified from genomic DNA of BY4741 by PCR, in a 20 μL mix containing 0.2 mM dNTP without dCTP, 5 μL of [α-32P]-dCTP (3,000 Ci/mmol, 10 mCi/mL; Perkin Elmer), 1 mM primer, 1x *Ex Taq* buffer and 1 unit of *Ex Taq* (TaKaRa). The mix was incubated at 94°C for 3 min, then cycled 35 times at 94°C for 20 sec, 51°C for 30 sec, and 72°C for 30 sec; unincorporated nucleotides were removed using ProbeQuant G-50 Micro Columns (GE Healthcare). Pre-hybridization was at 42°C for 1 h, heat-denatured probe was added, and the membrane was further incubated overnight at 42°C. The membrane was washed 1 × 15 min at 42°C with 2 × SSC, 0.1% SDS, then washed 2 × 15 min at 42°C with 0.1 × SSC, 0.1% SDS, and exposed to a phosphor screen. The radioactive signal was detected with a FLA7000 (GE Healthcare).

For hybridization with the ACT1 probe, which was done after probing for IGS1-F and IGS1-R transcripts, the membranes were first stripped in boiled 0.1% SDS for 30 min, and washed with 2 × SSC, 0.1% SDS.

### Genomic DNA preparation in plugs.

DNA plugs for PFGE analysis, ERC analysis, 2D analysis, and DSB analysis were prepared as previously described (14). For PFGE analysis and ERC analysis cells were cultured from a single colony for about 30 generations in YPD medium or in SC glucose media without uracil when a plasmid needed to be maintained. For 2D analysis and DSB analysis cells in the exponential growth phase had been grown in YPD medium to a density of 2 × 10^7^ cells/mL. 5 × 10^7^ cells were mounted in each plug of low-melting-temperature agarose. For PFGE analysis and ERC analysis, collected cells had been washed twice with 50 mM EDTA (pH 7.5). For 2D analysis and DSB analysis, sodium azide had been added to cells to a final concentration of 1 g/L, after which the cells were washed twice with 50 mM EDTA (pH 7.5). For each agarose plug, washed cells were resuspended in 33 μL of 50 mM EDTA (pH 7.5) and then mixed with 66 μL of a solution containing 8.3 mg/mL low-melting-point agarose SeaPlaque GTG (Lonza), 170 mM sorbitol, 17 mM sodium citrate, 10 mM EDTA (pH 7.5), 0.85% vol/vol β-mercaptoethanol, and 0.17 mg/mL Zymolyase 100 T (Nacalai). The solution was vortexed and poured into plug molds (Bio-Rad). Agarose plugs were solidified at 4°C. The plugs were treated with a solution containing 450 mM EDTA (pH 7.5), 10 mM Tris-HCl (pH 7.5), 7.5% vol/vol β-mercaptoethanol, and 10 mg/mL RNaseA (Macherey-Nagel) for 1 to 1.5 h at 37°C. Then, the plugs were incubated in a solution containing 250 mM EDTA (pH 7.5), 10 mM Tris-HCl (pH 7.5), 10 g/L SDS and 1 mg/mL Proteinase K (Merck Millipore) overnight at 50°C. Plugs incubated overnight were washed four times with 50 mM EDTA (pH 7.5) and stored at 4°C.

### Electrophoresis for ERC and DSB analysis.

Electrophoresis for ERC analysis was performed as previously described (37). Briefly, plugs cut into 5 mm width were separated using 4 g/L STAR Agarose (Rikaken) in 1x TAE at 1 V/cm at 4°C for 48 h. After 24 h of electrophoresis, the 1x TAE buffer was changed to fresh buffer.

For DSB analysis, electrophoresis was performed as previously described (14) with small modifications. Plugs were cut into 5 mm width, equilibrated with 100 μL of 1.5x NEB 3.1 buffer, and then equilibrated with 200 μL of 1x NEB 3.1 buffer. Then, after addition of 3 μL of BglII (50 U/μL) in 50 μL of 1x NEB 3.1 buffer, each plug was incubated at 37°C overnight. BglII digested DNA in the plugs was separated by 7 g/L Seakam LE Agarose (Lonza) in 1x TBE at 2 V/cm for 22 h.

### DNA transfer by Southern blotting.

After electrophoresis, the gel was stained in 200 μg/mL ethidium bromide (EtBr) to check separation of DNA and then soaked in 500 mL of 0.2 M HCl for 30 min and denatured in 500 mL of denaturation solution (0.5 M NaOH, 1.5 M NaCl) for 30 min at room temperature. The gel was neutralized in 500 mL of neutralization solution (1 M Tris-HCl pH 8.0, 1.5 M NaCl) for 30 min at room temperature and the DNA was capillary-transferred to Hybond-N+ (GE Healthcare) using 10 × SSC overnight. After the transfer, DNA was fixed with 120,000 μJ/cm^2^ in a Stratalinker (Stratagene, Model 1800), and the membrane was air-dried after washing in 5 × SSC.

### Hybridization of Southern blots.

Gel-purified fragments, amplified from genomic DNA of BY4741 by PCR using KOD Fx Neo (Toyobo) were used as templates to generate radioactive probes for Southern blotting using a Random Primer DNA Labeling Kit Ver.2 (TaKaRa). Briefly, 50 ng of DNA was heat-denatured, and incubated for 10 min at 37°C in a 25 μL mix containing 2 μL random primers, 2.5 μL 10x buffer, 2.5 μL dNTPs, 1 μL exo-free Klenow Fragment, and 5 μL [α-32P]-dCTP (3,000 Ci/mmol, 10 mCi/mL, Perkin Elmer). The mix was heat-denatured, and unincorporated nucleotides were removed using ProbeQuant G-50 Micro Columns (GE Healthcare).

Hybridization was performed as described previously (55). Briefly, the membrane was incubated in a hybridization bottle with 25 mL of hybridization buffer (10 g/L bovine serum albumin [BSA; Nacalai tesque, 01281-84], 0.5 M phosphate buffer [pH 7.2], 70 g/L sodium dodecyl sulfate [SDS], 1 mM EDTA [pH 8.0]) at 65°C for 1 h. Heat-denatured probe was added, and the membrane was further incubated over night at 65°C. After hybridization, the membrane was washed once for 30 min at 65°C with wash buffer 1 (2 × SSC, 1 g/L SDS), then washed twice for 30 min at 65°C with wash buffer 2 (0.1 × SSC, 1 g/L SDS). The washed membrane was sealed into a plastic film and exposed to a phosphorimaging screen which was scanned on a Typhoon FLA7000 (GE Healthcare).

### Pulse Field Gel Electrophoresis (PFGE) analysis.

PFGE analysis was performed as previously described (14). The plugs with genomic DNA were cut to 3 mm width and the DNA was separated over 1% agarose (Pulsed Field Certified Agarose, Bio-Rad) in 0.5x TBE. PFGE was performed on a Bio-Rad CHEF DR-III system in 2.2 L of 0.5x TBE with the following settings: 3.0 V/cm, run time = 68 h, included angle = 120°, initial switch time = 5 min, final switch time = 15 min, and ramping factor = linear).
